# Differentiated Entry or “Me-Too” Entry in Bertrand and Cournot Oligopoly

**DOI:** 10.1007/s11151-021-09822-1

**Published:** 2021-07-13

**Authors:** James A. Brander, Barbara J. Spencer

**Affiliations:** Sauder School of Business, University of British Columbia, Vancouver, BC V6T 1Z2 Canada

**Keywords:** Bertrand, Cournot, Entry, Horizontal product differentiation, Me-too products, D4, L1, L13

## Abstract

When would an oligopolistic entrant imitate an incumbent’s product (“me-too” entry), rather than horizontally differentiate? We allow an entrant's product choice to vary endogenously with the cost of product differentiation. Such endogenity of product differentiation significantly affects the comparison of Bertrand and Cournot duopoly. We find that if Bertrand entry occurs, products are differentiated, whereas there is a substantial region in which Cournot entry involves a homogenous product. Bertrand prices may be higher than Cournot prices; and, if product differentiation costs are low enough to induce Cournot differentiated entry, then Bertrand industry profit equals or exceeds Cournot industry profit.

## Introduction

A substantial amount of entry is by undifferentiated or “me-too” entrants, whose products are very similar to those of incumbent firms, as a quick trip around a grocery store or pharmacy will confirm.^1^ Differentiated entry is also common, however. Such differentiation may be vertical (quality-based), spatial (location-based), or horizontal (based on other characteristics). When using vertical or spatial models to analyze entry, differentiated entry is standard, as entrants typically choose quality levels or locations that differ from those of incumbents. In contrast, with horizontal entry in a non-spatial context, me-too entry is a significant possibility.

Our first main research objective is to determine when horizontal entry would occur and when such entry would be differentiated rather than me-too entry; we use a standard differentiated-product oligopoly model with quadratic utility. Another main objective is to determine the implications of endogenous product differentiation for the extent of product differentiation and for industry performance; we focus particularly on the comparison of Bertrand and Cournot competition. We vary the “effectiveness” of product differentiation investment—which is inversely related to the cost of differentiation—and then compare the results for Bertrand and Cournot entry.

We find that a Cournot entrant chooses not to invest in differentiation—and instead engages in me-too entry—for a significant range of product differentiation costs, while a Bertrand entrant always differentiates its product.^2^In part of this region of me-too Cournot entry, we find that the Bertrand price *exceeds* the Cournot price.^3^ When product differentiation costs are sufficiently low that the Cournot entrant does differentiate its product, a Bertrand entrant always differentiates its product by more, which results in a Bertrand industry profit that *equals or exceeds* Cournot industry profit.

The above results contrast with the commonly held belief that price and profit are generally lower under Bertrand than Cournot competition. However, this traditional understanding is based mainly on comparisons at a common level of product differentiation. Our results are explained by the much greater incentive for a Bertrand entrant to reduce head-to head competition by investing in differentiation. Another notable finding is that a decrease in the cost of product differentiation reduces consumer surplus in a Bertrand market. In a Cournot market, consumer surplus is unchanged in the region of me-too entry and increases thereafter. Despite these results, consumer surplus is always higher under Bertrand than Cournot competition.

Section 2 reviews the literature. Section 3 describes the basic model structure, and Sects. 4 and 5 develop the implications of horizontal product differentiation for Bertrand and Cournot entry, respectively. Section 6 provides comparative results, and Sect. 7 contains concluding remarks. Other than for Proposition 1, proofs are relegated to Appendices.

## Literature Review

Hotelling (1929) provides the classic analysis of spatial differentiation, and a large volume of research on spatial differentiation has followed. Spatial differentiation is an important form of horizontal product differentiation, but spatial differentiation raises some distinct issues. In the spatial literature, firms typically select a location without incurring location-specific entry costs. But there are significant exceptions—including Kishihara and Matsubayashi (2020)—who consider repositioning costs in a Hotelling framework.

There is also a large literature on vertical differentiation and entry, including: Shaked and Sutton (1982), Donnenfeld and Weber (1992), Hsu and Wang (2005), and many others. Seim (2006) provides an empirical study of vertical differentiation by oligopolistic entrants. It is possible to consider vertical differentiation in combination with location choice (as in Gabszewicz & Wauthy, 2012) or in combination with horizontal differentiation (as in Tremblay & Polasky, 2002). Vertical differentiation is empirically important in many contexts but we abstract from it here to focus on horizontal product differentiation.

The current paper is a substantially revised (and retitled) version of our earlier discussion paper: Brander and Spencer (2015a).^4^ Brander and Spencer (2015b) draws on the model in Brander and Spencer (2015a) to consider the implications of endogenous product differentiation for international trade. Liu et al. (2020) modify the model in Brander and Spencer (2015a) to examine a mixed oligopoly in which a private and public firm engage in price or quantity competition.

Lambertini and Rossini (LR) (1998) and Lin and Saggi (LS) (2002) also consider horizontal product differentiation in Bertrand and Cournot duopoly. Bertrand firms have a higher incentive to invest in both of these papers. LR allow a binary investment decision (invest or don't invest) and demonstrate a prisoner’s dilemma aspect to the decision. LS examine product and process R&D where product R&D results in horizontal product differentiation. Their model differs from our in several ways: Most importantly, LS assume that the first unit of R&D is costless, which rules out homogeneous products and therefore rules out "me-too" entry. Also, their R&D cost function is not designed to examine the effects of variation in differentiation cost.

The comparative properties of Bertrand and Cournot models have been addressed in a substantial literature. The basic finding—which is provided by Singh and Vives (1984), Cheng (1985), and Vives (1985), among others—is that if Bertrand and Cournot duopolies face the same conditions, the Bertrand industry would generate lower profits, lower prices, more consumer surplus, and more total surplus. However, Qiu (1997) shows that Cournot firms may invest more in cost-reducing R&D—which would possibly increase total surplus above the Bertrand level.

In this paper, we compare otherwise equivalent Bertrand and Cournot industries. As pointed out by a reviewer, we would not in practice expect to observe markets that differ only in this way. Presumably there is an underlying reason for whether the Bertrand or Cournot mode of conduct emerges—likely something that is related to technology, as in Kreps and Scheinkman (1983). If so, that underlying factor might also lead to different cost or demand conditions. Still, we share the commonly held view that focusing on the specific implications of mode of competition—holding other things constant—is valuable in understanding oligopoly behavior.

There is a body of literature in which the mode of conduct (Bertrand or Cournot) is regarded as endogenous, as in the classic treatment of Singh and Vives (1984). That literature typically assumes that firms can sign binding price or quantity contracts, which allows them to choose the mode of competition. Such contracts are not uncommon but are far from the norm. We do not take a position on the empirical significance of endogenous mode-of-competition models. Our product differentiation decision could be subsequent to an initial stage in which firms choose the mode of conduct, or the mode of conduct could be exogenous.

## Modelling Preliminaries

We use a two-stage game to examine potential entry into a market with an incumbent firm that produces a pre-existing product. In the first stage, a potential entrant considers whether to enter and, if so, how much to invest in product differentiation. If the entrant copies the incumbent’s product—which gives rise to homogeneous products—no differentiation investment is required. Development of its own differentiated variety requires a stage-1 investment by the entrant. There is also a small fixed entry cost *E* that is required—whether or not product differentiation occurs. In stage 2, the entrant and the incumbent compete on the basis of either Cournot or Bertrand competition. If there is no entry, the incumbent has a monopoly in stage 2.

The incumbent’s product characteristics are fixed. The entrant chooses its stage 1 differentiation investment (which may be zero) to maximize its profit; the entrant fully anticipates the effect on its variable profit in stage 2 in accordance with a subgame perfect Nash equilibrium.

We assume a standard quadratic utility function. Letting *x*_*1*_ and *x*_*2*_ represent the output of the incumbent and entrant, respectively, the aggregate or representative utility function is

1 $$U = a\left( {x_{1} + x_{2} } \right) - \left( {x_{1}^{2} + x_{2}^{2} } \right)/2 - sx_{1} x_{2} + M,$$

where *M* denotes the consumption of a numeraire good. Because *U* is additively separable in *M*, there are no income effects of demand. The parameter *s* represents the degree of substitutability between the products *x*_*1*_ and *x*_*2*_. Goods *x*_*1*_ and *x*_*2*_ can range from being perfect substitutes (homogeneous) at *s* = 1 to being totally unrelated at *s* = 0, in which case each firm has a monopoly with respect to its own good. We do not examine complementary products (*s* < 0). If we let *p*_1_ and *p*_2_ represent prices, from (1), inverse demand curves are normalized to have a slope of − 1. If *s* < 1, then

2 $$p_{1} = a{-}x_{1} {-}sx_{2} ;\quad p_{2} = a{-}x_{2} {-}sx_{1} .$$

If *s* = 1 and prices differ, consumers buy only from the low-price firm. If both firms charge the same price, we adopt the standard convention that they sell the same quantity.

It is convenient to define a parameter *v* ≡ 1 − *s* (*v* for “variety”) to measure product differentiation. Holding quantities *x*_*1*_*, x*_*2*_, and *M* constant, we obtain ∂*U*/∂*v* =  − ∂*U*/∂*s* = *x*_*1*_*x*_*2*_ > 0 from (1). Thus, consumers have a taste for variety: For given levels of consumption, an increase in product differentiation *v* makes consumers better off. As a result, product differentiation expands the market and also increases market power.

Product differentiation by the entrant increases consumer willingness to pay, but this differentiation can be achieved only by investing an amount *k* in product development costs. The relationship between *k* and the values of *s* and *v* is assumed to take the following equivalent forms, where β denotes a strictly positive parameter:

3 $$s\left( {k;{\upbeta }} \right) = e^{{ - {\upbeta }k}} ;\quad ~v\left( {k;{\upbeta }} \right) = 1 {-}e^{{ - {\upbeta }k}} .$$

The parameter β can be interpreted as a measure of the effectiveness of the entrant’s investment—*k*—in creating product differentiation. From (3), investment increases product differentiation, but at a decreasing rate:

4 $$v^{\prime } \left( k \right) = - s^{\prime } \left( k \right) = {\upbeta }s > 0;\quad v^{{\prime \prime }} \left( k \right) = - s^{{\prime \prime }} \left( k \right) = - {\upbeta }^{2} s < 0.$$

If *k* = 0, then β = *v*′(0) from (4), so β is equal to the increase in product differentiation that stems from the first unit of investment. For brevity, we sometimes refer to β as “investment effectiveness”.

For any given positive value of *k*, it follows from (3) that product differentiation *v* is greater if β is larger. Correspondingly, the cost *k* of achieving any given value of *v* is smaller if β is larger. Thus, the value of β is inversely related to the investment cost that is required to achieve any given level of product differentiation.

If the entrant invests nothing in product differentiation (*k* = 0) then, from (3), the products are homogeneous (*s* = 1 and *v* = 0). The other extreme—in which the products are independent (*s* = 0 and *v* = 1)—is approached only in the limit as investment *k* or investment effectiveness β approach infinity. Thus, strict monopoly does not occur for any finite value of *k* or *β*. This contrasts with the ("uncovered") Hotelling version of product differentiation in which firms can move sufficiently far apart that strict monopoly occurs. In the context of a comparison of Bertrand with Cournot oligopoly, abstracting from a strict monopoly corner solution conveniently avoids a special case of limited interest.

Also, as a practical matter, there is little difference between strict monopoly and near-monopoly, and near-monopoly is achieved at moderate levels of differentiation investment. Near monopoly would include products with a slight interaction even though we may think of them as independent, as with aspirin and cortico-steroids, or electric cars and battery-assisted bicycles. Also, we view it as plausible that—starting from a base in which an entrant could produce a me-too product—differentiation investment is unlikely to make the products completely independent.

## Bertrand Competition

### Second Stage: Pricing Decisions

Using backward induction, we now solve for the second-stage Bertrand equilibrium prices and output conditional on the degree of substitutability—*s*—and show how the equilibrium changes with *s*. We later consider the stage-1 entry and differentiation decision. Letting subscripts *i* = 1, 2 refer to the incumbent and entrant respectively, variable profits at stage 2 are given by *Φ*_*i*_ ≡ (*p*_*i*_ − *c*)*x*_*i*_, where the constant—*c*—represents the production marginal cost that is the same across firms and is unaffected by *k*. If entry occurs in stage 1, then in stage 2 each firm maximizes its variable profit with respect to its own price, taking the other firm’s price as given. Each firm also treats *k* and therefore *s* as fixed, as those variables are predetermined in stage 1.

For exogenous product differentiation, the properties of this model are well-known, and those same properties apply to our stage 2 solution. Using superscript *B* to identify variables and functions in the Bertrand version of our model, each firm sets the same price—*p*^*B*^(*s*)—which leads to a common output for each firm—*x*^*B*^(*s*)—and a common variable profit—*Φ*^*B*^(*s*)—as follows:

5 $$\begin{aligned} p^{B} & = p^{B} \left( s \right) = \left[ {\left( {a{-} c} \right)\left( {1 {-}s} \right)/\left( {2{-}s} \right)} \right] + c; \\ x^{B} & = x^{B} \left( s \right) = \left( {a {-} c} \right)/\left[\left( {2{-} s} \right)\left( {1 + s} \right)\right]; \\ \Phi ^{B} & = \Phi ^{B} \left( s \right) = \left( {1{-} s^{2} } \right)\left( {x^{B} \left( s \right)} \right)^{2} . \\ \end{aligned}$$

We impose *a* > *c* to ensure that output is positive.

If *s* = 1 (*v* = 0), the expressions in (5) yield the standard Bertrand homogeneous product outcomes. These expressions also imply that decreases in *s* (increases in *v*) cause Bertrand prices and variable profits to rise (see (A2)). In the limit as *s* approaches 0, the products approach becoming unrelated.

Interestingly, the response of output to increased product differentiation is “U-shaped”. An increase in substitutability (reduction in differentiation) affects output as follows:

6 $${\text{d}}x^{B} /{\text{d}}s = x^{B} \left( s \right)\left( {2s{-}{1}} \right)/\left[ {\left( {2{-}s} \right)\left( {1 + s} \right)} \right].$$

As *v* = 1 − *s*, it follows that d*x*^*B*^/d*v* =  − d*x*^*B*^/d*s* which, using (6), is negative if *v* < ½, zero if *v* = ½, and positive if *v* > ½. This leads to Proposition 1:

#### **Proposition 1**: Effects of increased product differentiation on Bertrand output

*If product differentiation v increases, Bertrand outputs fall if 0 ≤ v < ½, reach a minimum at v = 1/2, and then rise for ½ < v < 1.*

As products change from being identical to being slightly differentiated, output initially falls because Bertrand firms sharply increase price. As *v* gets larger, further increases in differentiation have a smaller effect on price and the market expansion effect of increasing variety eventually (for *v* > ½) dominates, which leads to output increases*.*^5^

### First Stage: Investment in Product Differentiation and Entry

In the first stage, the potential Bertrand entrant determines its profit-maximizing level of investment, *k*, and decides whether or not to enter, taking into account the entry cost, *E*. The potential entrant anticipates the effects of *k* on the second-stage equilibrium variable profit—*Φ*^*B*^(*s*(*k;*β))—that are due to product differentiation. The entrant’s stage-1 profit from entry is denoted by

7 $${\uppi}^{B} = {\uppi }^{B} \left( {k;{\upbeta }} \right) \equiv \Phi ^{B} \left( {s\left( {k;\beta } \right)} \right){-}k{-}E.$$

The profit-maximizing level of investment satisfies the first-order condition—dπ^*B*^(*k*)/d*k* = 0—for an interior solution; but for the purposes of considering entry, the corner solution where *k* = 0 is important. From (7), the first-order condition for the choice of *k* is

8 $${\text{d}}{\uppi }^{B} \left( k \right)/{\text{d}}k = \left( {{\text{d}}\Phi ^{B} \left( s \right)/{\text{d}}s} \right)s^{\prime} \left( k \right){-}1 \le 0\left( { = 0\,\,{\text{if}}\,\,k > 0} \right),$$

where *s*′(*k*) =  − βs from (4) and d*Φ*^*B*^(*s*)*/*d*s* =  − 2(*x*^*B*^(*s*))^2^(1 − *s* + *s*^2^)/(2 − *s*) from (A2) in Appendix 1. We establish the strict concavity of π^*B*^(*k*) in Lemma 1 (see Appendix 1), which ensures a unique profit maximum at a value of *k* = *k*^*B*^ that satisfies (8).

Entry takes place if the profit from entry with *k* chosen optimally is positive or zero. If the firm does not enter, it does not pay *E*, and its profit is zero. If the firm enters, but does not invest (*k* = 0), then products are homogeneous and variable profit under Bertrand competition is zero. The cost *E* prevents entry in this case. In addition to being realistic, the entry cost allows us to rule out me-too Bertrand entry and focus on differentiated entry.

To avoid additional cases of not much interest in this context, we assume that *E* is positive but very small:

9 $$E = 0.0001\left( {a{-}c} \right)^{2} .$$

We use this value of *E* later in simulations.

Proposition 2 sets out the conditions that determine Bertrand investment and entry.

#### **Proposition 2**: Characteristics of Bertrand investment and entry

(i) *Entry under Bertrand competition requires investment in product differentiation. For any given value of β, the equilibrium level of investment, k = k* ^*B*^ *, is unique and finite.*
(ii) *A necessary condition for Bertrand entry is β* > *2*/*(a* − *c)*^*2*^*. For lower levels of β, product differentiation is not profitable (k*^*B*^ = *0), and entry would not occur.*
(iii) *For E* = *0.0001(a* − *c)*^*2*^*, Bertrand entry takes place if and only if β* ≥ *β*^*E*^* ≡ 2.09/(a* − *c)*^*2*^*.*

#### *Proof*

See Appendix 1.

From Proposition 2(ii), investment in product differentiation requires investment effectiveness β to strictly exceed a threshold level—2/(*a* − *c*)^2^—that depends on the magnitude of the difference between the demand intercept, *a*, and marginal cost, *c*. The stronger is demand, the less effective investment needs to be to justify entry and product differentiation. As *E* is positive (although very small), β must exceed 2.09/(*a* − *c*)^2^ to generate entry.

## Cournot Competition

### Second Stage: Quantity Decisions

We now consider Cournot competition in the post-entry stage. The incumbent and the entrant each set output to maximize their variable profit; they each take the output of the other firm as given, and they each also treat *k*—which is committed by the entrant in stage 1—and therefore *s* and *v*, as fixed.

Using superscript *C* to identify variables and functional relationships that are associated with Cournot competition, we show that the common output, price, and variable profit in the second stage Cournot equilibrium are as follows:

10 $$\begin{aligned} x^{C} & = x^{C} \left( s \right) = \left( {a{-}c} \right)/\left( {2 + s} \right); \\ p^{C} & = p^{C} \left( s \right) = \left[ {\left( {a{-}c} \right)/\left( {2 + s} \right)} \right] + c; \\ \Phi ^{C} & = \Phi ^{C} \left( s \right) = \left( {x^{C} \left( s \right)} \right)^{2} . \\ \end{aligned}$$

If *s* = 1 (*v* = 0), then the expressions in (10) reduce to the standard homogenous product Cournot levels. Also, recalling that *v* = 1 − *s*, it is easy to show that increases in *v* cause Cournot output, price, and variable profits to rise. In the limit as *v* approaches 1, each firm produces the monopoly output in its submarket—just as in the Bertrand case.

### First Stage: Investment in Product Differentiation and Entry

In the first stage, the potential Cournot entrant correctly anticipates that, if it enters, variables will take on the Cournot equilibrium values in the second stage; and consequently the entrant will set its investment—*k* ≥ 0—to maximize its profit: π^*C*^(*k*;β) = *Φ*^*C*^(*s*(*k*;*β*)) − *k* − *E*. Maximizing profit yields the following first-order condition:

11 $${\text{d}}{\uppi }^{C} \left( k \right)/{\text{d}}k = \left( {{\text{d}}\Phi ^{C} \left( s \right)/{\text{d}}s} \right)s^{\prime } \left( k \right){-}1 \le 0\left( { = 0\,\,\,{\text{if}}\,\,\,k > 0} \right),$$

where *s*'(*k*) =  − β*s* and d*Φ*^*C*^(*s*)/d*s* =  − 2(*x*^*C*^(*s*))^2^/(2 + *s*) from (A5) in Appendix 2. As is established in Lemma 2 (see Appendix 2), π^*C*^(*k*) is strictly concave, which ensures a unique profit maximum at *k* = *k*^*C*^ that satisfies (11).

Proposition 3 sets out the conditions that determine investment and entry in the Cournot case. If an entrant does not differentiate its product (*k* = 0 and *s* = 1), then from *Φ*^*C*^(1) = (*a* − *c*)^2^/9 (see (10)) and *E* as in (9), its profit from me-too entry—π^*C*^(0) = (*a* − *c*)^2^/9 − *E*—is strictly positive. Consequently, under our assumptions that firms face the same marginal cost and *a* > *c*, entry always occurs in the Cournot case. The entrant invests in product differentiation only if such investment is profitable:

#### **Proposition 3**: Characteristics of Cournot investment and entry

(i) *A potential Cournot entrant always enters, but may choose not to invest in differentiation. For any given value of β, the equilibrium level of investment k* ^*C*^ *is unique and finite.*
(ii) *If β* ≤ *13.5/(a* − *c)*^*2*^*, then k*^*C*^ = *0, and the entrant imitates the incumbent’s product (me-too entry). If β* > *13.5/(a* − *c)*^*2*^*, then the entrant differentiates its product, and thus sets k*^*C*^ > *0.*

#### *Proof*

See Appendix 2.

## Comparison of Bertrand and Cournot Competition

### Comparative Conditions for Product Differentiation

Propositions 2 and 3 focus on the thresholds at which entry and investment take place as determined by investment effectiveness β. Proposition 4 compares these threshold conditions and examines the relative magnitudes of investment:

#### **Proposition 4**: Bertrand and Cournot investment comparisons

(i) *If β* ≤ *2/(a* − *c)*^*2*^*, then product differentiation is not profitable for either Cournot or Bertrand entrants. A potential Bertrand entrant would not enter. A potential Cournot entrant would enter, but would engage in me-too (undifferentiated) entry.*
(ii) *If β*^*E*^ ≤ *β* ≤ *13.5/(a* − *c)*^*2*^* where β*^*E*^* ≡ 2.09/(a* − *c)*^*2*^*, then the Bertrand entrant differentiates its product, whereas the Cournot entrant engages in me-too (undifferentiated) entry: k*^*B*^ > *k*^*C*^ = *0.*
  *If β* = *13.5/(a* − *c)*^*2*^*, then v*^*B*^ = *0.608, which implies that 60.8% of potential Bertrand product differentiation as measured by v is achieved in the region of Cournot me-too entry.*
(iii) *If β* > *13.5/(a* − *c)*^*2*^*, then an entrant*—*whether Bertrand or Cournot*—*invests in product differentiation, but a Bertrand entrant invests more than does a Cournot entrant: k*^*B*^ > *k*^*C*^ > *0.*

#### *Proof*

See Appendix 3.

As Proposition 4 shows, endogenous product differentiation results in greater investment and product differentiation under Bertrand than under Cournot competition. Furthermore, the threshold value of β at which differentiation begins (*13.5/(a* − *c)*^2^) is quite large in that 60.8% of potential Bertrand product differentiation—as measured by *v*—occurs in the range of β below this threshold. These differences in product differentiation incentives play a fundamental role in the price and profit comparisons that will be considered in the next section.

Proposition 5 concerns the effects of investment effectiveness β on investment and product differentiation, prices, and profit: For both Bertrand and Cournot competition, investment *k* is initially increasing in β, reaches a maximum, and then declines. At high levels of product differentiation, it becomes profitable for the entrant to save costs by investing less. Even if *k* is reduced as β is increased, product differentiation nevertheless continues to increase. Prices and profits for both the entrant and the incumbent also increase as β increases:

#### **Proposition 5**: Effects of an increase in β:

*For both Bertrand and Cournot competition, starting from a level of β that is just sufficient to induce differentiated entry, further increases in β:*

(i) *Initially increase and then reduce investment in product differentiation;*
(ii) *Increase product differentiation; and*
(iii) *Increase prices and the profits of the incumbent and the entrant.*

#### *Proof*

See Appendix 3.

We illustrate the effects of variations in investment effectiveness β using diagrams that are generated in Python 3.7. The simulations assume specific parameter values *a* = 1 and *c* = 0, which imply *E* = 0.0001 from (9).^6^ Lemma 3 (see Appendix 4) establishes that similar diagrams apply for any *a* and *c*—provided that *a* > *c*. For any given level of product differentiation, investment, price, and profit are multiplied by (*a* − *c*)^2^ and β is divided by (*a* − *c*)^2^.

**Fig. 1:**
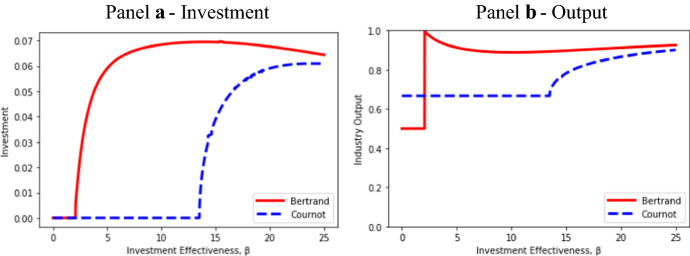
Effects of *β* on investment and output

Figure 1 illustrates the effects on investment and output as β ranges from 0 to 25 along the horizontal axis. As is shown in Panel a, for low levels of β (high differentiation costs), neither Bertrand firms nor Cournot firms differentiate their products. There is no Bertrand entry, so industry output is the incumbent’s monopoly output, as is shown in Panel b. In contrast, the Cournot firm enters, which results in Cournot industry output at the duopoly homogeneous product level. From Proposition 4, Bertrand entry with a differentiated product requires β to be at or above the threshold level: β^E^ = 2.09/(*a* − *c*)^2^ (= 2.09 in this simulation). For a Cournot entrant, the threshold is much higher: at β > 13.5 in this simulation.

As expected from the “U shaped” relationship between product differentiation and output under Bertrand competition (Proposition 1), Panel b of Fig. 1 shows Bertrand industry output as falling for an initial range of β subsequent to entry. Bertrand output rises slightly once *v*^*B*^ > ½, which corresponds to β > 10.125 in this simulation. Cournot output is constant or increasing in β; but, as we show in Proposition 6, Cournot output never reaches the Bertrand level.

#### **Proposition 6**: Bertrand and Cournot Output Comparison

*If there is Bertrand entry, output is always higher under Bertrand than Cournot competition.*

#### *Proof*

See Appendix 5.

As is illustrated in Panel a of Fig. 1, if entry occurs, Bertrand investment in product differentiation always exceeds Cournot investment (Proposition 4). The large region of me-too (undifferentiated) Cournot entry where there is Bertrand investment, magnifies the difference between investment levels and therefore the difference in the extent of product differentiation.

After the investment threshold is reached, investment initially increases with β, reaches a maximum, and then declines under both modes of competition (Proposition 5). But there are major differences between the investment paths. In particular, from Panel a of Fig. 1, Bertrand investment begins to decline while Cournot investment is still increasing. This result helps to explain the mechanism by which Bertrand and Cournot output—and also price and profit—become very similar as β becomes large. In the limit as β increases, product differentiation approaches being costless. An entrant of either the Bertrand or Cournot type would then completely differentiate its product (*v* = 1 or *s* = 0) so as to result in two monopolies in disjoint submarkets.

### Endogenous Product Differentiation and the Intensity of Competition

It is well-known that if products are homogeneous, Bertrand competition is more intense than Cournot competition, with lower prices and profits. This insight generalizes to any *common* level of product differentiation short of being completely unrelated—at least for our demand structure. It is therefore not surprising that the competition-softening effect of product differentiation is of greater marginal benefit to Bertrand firms.

As can be shown from (A10), (A2), (A5), and *s* = 1 − *v*, for a *common* level of *v*, an increase in *v* raises price and variable profit by more under Bertrand than Cournot competition:

12 $${\text{d}}p^{B} /{\text{d}}v > {\text{d}}p^{C} /{\text{d}}v\,\,{\text{and}}\,\,{\text{d}}\Phi ^{B} /{\text{d}}v > {\text{d}}\Phi ^{C} /{\text{d}}v.$$

Condition (12) explains why a Bertrand entrant always invests more (Proposition 4(iii)) and why endogenous product differentiation has important effects on relative prices and profits.

**Fig. 2:**
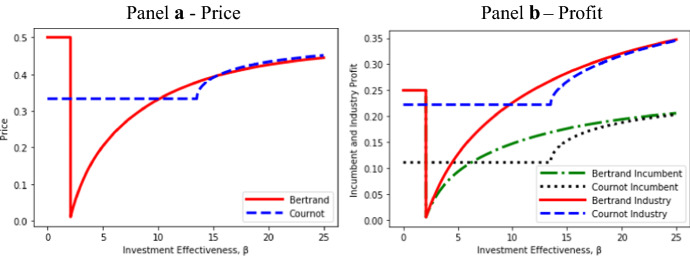
Effects of *β* on prices and profit

Using the parameter values *a* = 1 and *c* = 0 again to generate diagrams, Fig. 2 illustrates the effects of investment effectiveness β on Bertrand and Cournot prices and profits.

For very low values of β, there is no Bertrand entry so the incumbent charges the monopoly price (Panel a) and earns monopoly profits (Panel b), which exceed the Cournot duopoly price and profit. In this range (potential) Bertrand competition is “less competitive”. At values of β immediately above this range, we get the conventional result that Bertrand firms charge a lower common price and earn lower profits than do Cournot firms. However, as β is further increased, the Bertrand price actually exceeds the Cournot price for a range of β. Bertrand incumbent and industry profit also rise above Cournot incumbent and industry profit.

We emphasize that the regions of β in which the Bertrand price or profit exceed the Cournot price or profit are not an artifact of the specific values *a* = 1 and *c* = 0 used in Fig. 2. As implied by Lemma 3 of Appendix 4, these regions exist for any feasible values of *a* and *c*. The general conditions giving rise to a higher Bertrand price are established in Proposition 7:

#### **Proposition 7**: Bertrand and Cournot price comparisons:

*There is a region of β*—*β* ∈ (10.125/(*a* − *c*)^2^, 15.25/(*a* − *c*)^2^]—*where the Bertrand price exceeds the Cournot price. This includes that part of the region of Cournot me-too entry in which more than 50% of Bertrand product differentiation as measured by v*^*B*^ > *½ has been achieved.*

#### *Proof*

See Appendix 5.

For any given form of competition (Bertrand or Cournot), greater product differentiation increases price in our setting because consumers value variety. To understand why Bertrand investment in product differentiation can be sufficiently large to raise the Bertrand price above the Cournot price, we use (5) and (10) to obtain

13 $$p^{B} > p^{C} \,\,{\text{if and only if}}\,\,s^{B} < s^{C} /\left( {1 + s^{C} } \right).$$

In the region of Cournot me-too entry, we have *s*^*C*^ = 1, and it follows from (13) that *p*^*B*^ > *p*^*C*^ if and only if *s*^*B*^ < ½ or *v*^*B*^ > ½ as shown in Proposition 7. Since an increase in β raises *p*^*B*^, but *p*^*C*^ is unchanged, me-too Cournot entry enhances the potential for *p*^*B*^ > *p*^*C*^. As can be seen from Panel a of Fig. 2, this result applies for β ∈ (10.125, 13.5]. At β = 13.5 (corresponding to *s*^*B*^ = 0.392), the gap between *p*^B^ and *p*^C^ is at a maximum.

For β > 13.5 in Panel a of Fig. 2, differentiated Cournot entry causes the Cournot price to rise much more rapidly than the Bertrand price, but from the continuity of product differentiation and price in β, *p*^*B*^ remains above *p*^*C*^ for a further range of values of β: for β ∈ (13.5, 15.25]. Insight for this result is provided by Panel a of Fig. 1, which shows that at β = 13.5, Bertrand investment in product differentiation is very close to its maximum, whereas Cournot investment increases sharply from zero as β is increased above 13.5. Even without additional investment, product differentiation and price increase with β (see Proposition 5), but the plateauing of investment and its eventual fall causes the Bertrand price increase to slow.

As shown in Panel b of Fig. 2, Bertrand incumbent and industry profit can exceed Cournot levels. Since output is higher under Bertrand competition (Proposition 6), it follows immediately that, in the region where price is higher, the variable profit of each firm is also higher. This includes the region that encompasses both me-too entry and *v*^*B*^ ≥ ½ (see Proposition 7). More surprisingly, Bertrand variable profit is higher for the entire region (β > 13.5) in which the Cournot entrant invests. Also, for β > 13.5, Bertrand industry profit equals or exceeds Cournot industry profit.

These profit results are set out in Proposition 8. Proposition 8(i) concerning variable (incumbent) profit is proved analytically, but numerical methods are used to incorporate the cost of investment and compare industry profits in Proposition 8(ii). The results apply for any *a* > *c*:

#### **Proposition 8**: Bertrand and Cournot profit comparisons:

*If the Cournot entrant differentiates its product (β > 13.5/(a − c)* ^*2*^ *), then:*

(i) *Variable profit is higher under Bertrand than Cournot competition;*
(ii) *Bertrand industry profit equals or exceeds Cournot industry profit.*

#### *Proof*

See Appendix 6.

The fundamental explanation for a higher Bertrand price or profit (Propositions 7 and 8) is that product differentiation is much more valuable at the margin to Bertrand than to Cournot firms (see (12)). Specifically, the additional Bertrand profit from an initial differentiation in products from the homogeneous level (*s* = 1) is more than six times the additional Cournot profit. Such product differentiation increases the Bertrand price so sharply that output falls: The Bertrand price increases nine times the initial increase in the Cournot price.^7^ This large difference in investment incentives also explains why—starting from *s* = 1—product differentiation becomes attractive for a Bertrand entrant at a much lower level of β than is true for a Cournot entrant.

**Fig. 3:**
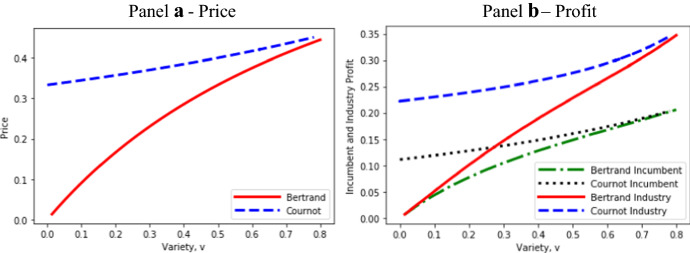
Effects of product variety on Bertrand and Cournot prices and profits

Figure 3 compares prices and profits under Bertrand and Cournot competition for a common level of product differentiation or variety, much as in the conventional analysis where product differentiation is exogenous. The Figure uses *a* = 1, *c* = 0 (as before) and values of β that are large enough to induce Bertrand entry (β > 2.09).

As expected, Fig. 3 shows that for the same level of product variety, Cournot prices exceed Bertrand prices (Panel a) and Cournot profits exceed Bertrand profits (Panel b). However, as was illustrated by Panel a of Fig. 1, Bertrand and Cournot entrants would *not* invest the same amount in product differentiation, and therefore Bertrand and Cournot industries never have the same level of variety if their cost and demand parameters are identical. It follows that if product differentiation is endogenous, the assumption of exogenous and equivalent levels of product differentiation can lead to misleading profit and price comparisons of Bertrand and Cournot competition.

**Fig. 4:**
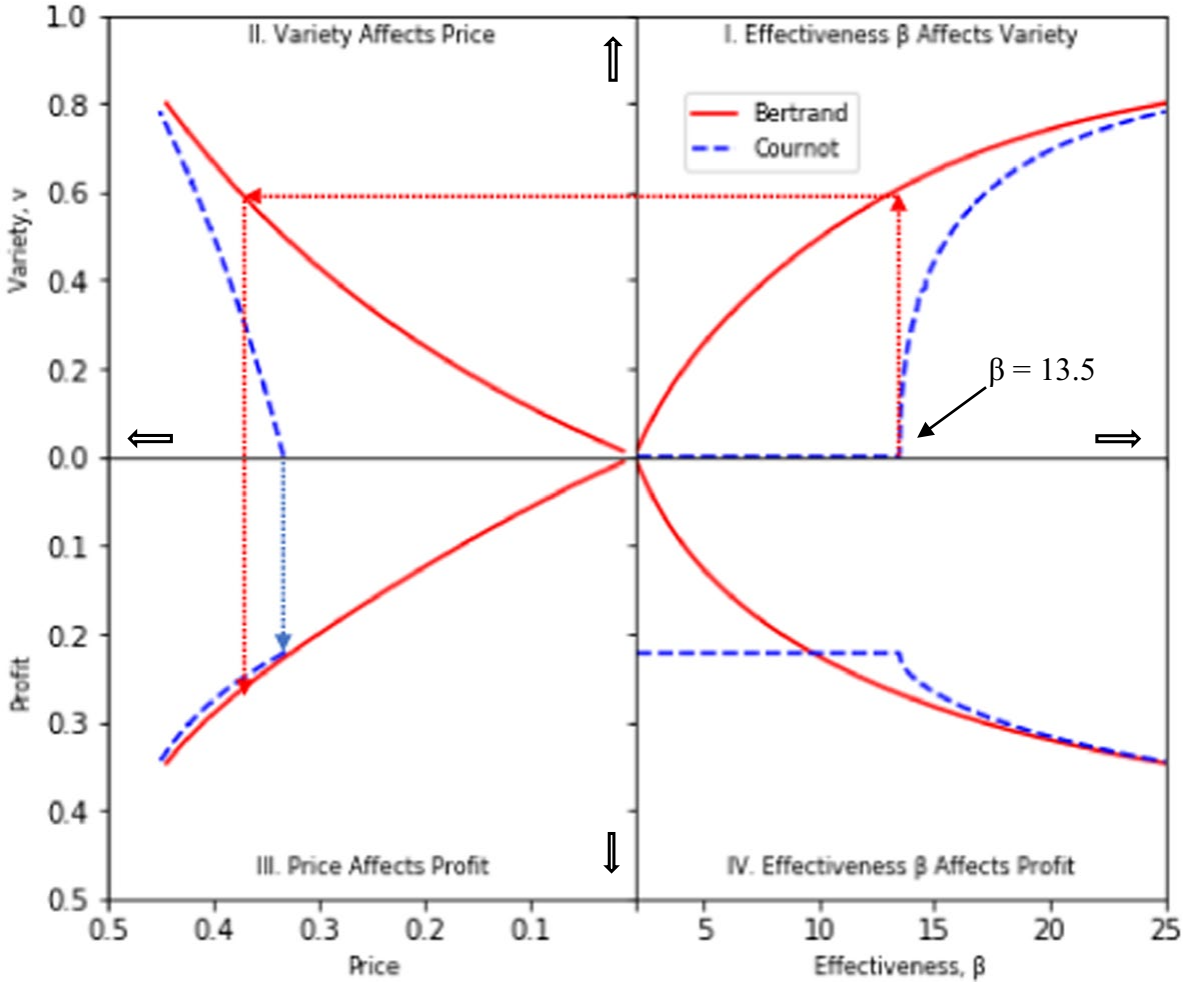
Bertrand and Cournot comparisons in a four-quadrant diagram

We next use Fig. 4—a four-quadrant diagram that relates β, variety, price, and profit—to illustrate the forces at work in our model and to show how endogenous product differentiation compares with the conventional assumption of fixed or exogenous differentiation.

We again assume that *a* = 1 and *c* = 0, and we use a solid line for the Bertrand model and a dashed line for the Cournot model. Quadrant 1 (upper right) shows the relationship between β and the resulting amount of product differentiation (variety) *v*. The dotted lines illustrate the case of β = 13.5: the critical value of β above which a Cournot entrant chooses to differentiate. At β = 13.5, a Cournot entrant imitates the incumbent’s product (*v*^*C*^ = 0), whereas a Bertrand entrant has a high level of product differentiation (*v*^*B*^ = 0.608). For β > 13.5, variety increases much more sharply under Cournot than under Bertrand competition, but never quite catches up to the Bertrand level.

Quadrant II (upper left) of Fig. 4 illustrates the relationship between variety and price as in Panel a of Fig. 3, but price is now measured from right-to-left along the horizontal axis. For any common value of *v* (along the vertical axis), it can be seen that the Cournot price exceeds the Bertrand price. If *v* is endogenous, then at β = 13.5, the Cournot price (at *v*^*C*^ = 0) is given by the intercept of the dashed line on the horizontal axis. The corresponding Bertrand price, as shown by the dotted horizontal line at *v* = 0.608 extended into Quadrant II, exceeds the Cournot price.

The relationship between price and industry profit is illustrated in Quadrant III where profit is measured downward along the vertical axis. For β = 13.5, the dotted lines that extend down from Quadrant II into Quadrant III show that, at the higher Bertrand price, Bertrand industry profit exceeds Cournot industry profit. If the prices that are associated with a common value of *v* in Quadrant II are linked to profit in Quadrant III, the price advantage from Cournot competition is sufficient to ensure that Cournot profit exceeds Bertrand profit.

Quadrant IV shows the relationship between β and industry profit as an “inverted” version of the same relationship that was shown in Panel b of Fig. 2. The constant Cournot profit level for β ≤ 13.5 corresponds to the horizontal part of the dashed line in Quadrant IV. For Bertrand profit to be higher, it is sufficient that β > 13.5.

A further interesting insight from Quadrant II is that the range of possible prices is much less for Cournot than for Bertrand competition. This is apparent from the much smaller range of support of the dashed Cournot price line on the horizontal axis than the corresponding range of support of the solid Bertrand price line. As can be seen from Quadrant III, a similar result applies to variation in profits.

### Endogenous Product Differentiation and Consumer Surplus

Endogenous horizontal product differentiation also affects consumer surplus. Letting *G*^*j*^(*s*) *≡ U*^*j*^ − *2p*^*j*^*x*^*j*^(*s*) − *M* denote consumer surplus (or “gain”), and using (1) and *p*^j^ = *a* − (1 + *s*)*x*^j^(*s*) from (2), we can express consumer surplus—which is evaluated at *s*^*j*^ ≡ *s*(*k*^*j*^;β)—as

14 $$G^{j} \left( {s^{j} } \right) = \left( {1 + s^{j} } \right)\left( {x^{j} \left( {s^{j} } \right)} \right)^{2} \quad {\text{for}}\,\,j = B,C.$$

Proposition 9 concerns the effects of an increase in investment effectiveness—β—on consumer surplus, and also on total surplus, under the two modes of competition. An increase in β increases product differentiation, which has two opposing effects on consumer surplus: Consumers value variety, so for the same price and output, consumer surplus tends to increase with product differentiation; but prices also increase. The outcome varies with the mode of competition:

#### **Proposition 9**: Effects of β on consumer and total surplus:

(i) *For Bertrand competition, if β is sufficiently large to induce entry, then an increase in β reduces consumer surplus. For total surplus to increase in β, it is sufficient that v*^*B*^ = *1* − *s*^*B*^ ≥ *½, which applies for β* ≥ 10.125/(*a* − *c)*^*2*^.
(ii) *For Cournot competition, an increase in β has no effect on consumer surplus or total surplus in the region of me-too entry; but once investment takes place (β* > *13.5/(a* − *c)*^*2*^*), consumer surplus and total surplus are increasing in β*.
(iii) *For any given value of β, consumer surplus is higher under Bertrand than Cournot competition. Total surplus is higher under Bertrand than Cournot competition if the Cournot entrant differentiates its product*.

#### *Proof*

See Appendix 6.

Proposition 9(i) and (ii) demonstrate an important difference between Bertrand and Cournot competition: Once Bertrand entry occurs, an increase in β causes a sufficient rise in the Bertrand price that consumer surplus falls. The increase in market power more than offsets consumer gains from expanded variety. This contrasts with the Cournot case where, if the entrant differentiates—β > 13.5/(*a* − *c*)^2^—an increase in β increases consumer surplus. The Cournot price starts from a higher base; and once product differentiation takes place, the resulting relatively modest price increase is not enough to offset the gains from increased variety. Despite these results, Proposition 9(iii) shows that consumer and total surplus are higher under Bertrand competition.

**Fig. 5:**
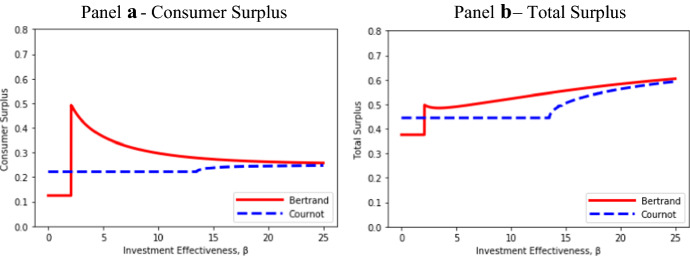
Effects of *β* on consumer and total surplus

Figure 5 illustrates the contrasting effects of β on consumer surplus and total surplus under Bertrand and Cournot competition. As in previous figures, we assume *a* = 1 and *c* = 0.

For low levels of β, consumer surplus, which is shown in Panel a of Fig. 5, rises very sharply as Bertrand entry takes place and the incumbent ceases to be a monopolist. With very little product differentiation, the Bertrand price is close to marginal cost, and consumer surplus is close to its maximum. As β increases beyond the point of Bertrand entry, there is a significant rise in the Bertrand price (see Fig. 2) and some initial reduction in Bertrand output (see Fig. 1)—both of which tend to reduce consumer surplus. Bertrand consumer surplus continues to fall as β becomes large, albeit more slowly, because price continues to rise and output increases only slightly.

After an initial slight decline, Bertrand total surplus increases in β as shown in Panel b of Fig. 5. This is because there is a rapid increase in Bertrand industry profit (see Fig. 2) that soon more-than-offsets the decline in consumer surplus.

In the Cournot regime, there is no effect of β in the range of me-too entry. Once product differentiation takes place, consumer surplus increases only slightly with β, because the consumer benefit from additional variety is almost fully offset by the increase in the Cournot price. In this region, Cournot industry profit increases more sharply than does consumer surplus (see Fig. 2), which gives rise to a larger increase in total surplus than in consumer surplus, as was shown in Panel b of Fig. 3.

Allowing for endogenous product differentiation, consumer surplus is always higher under Bertrand than Cournot competition (see Proposition 9(iii)). This may be puzzling given the range of β—β ∈ (10.125, 15.25]—in Fig. 2, where the Bertrand price strictly exceeds the Cournot price. These results are consistent because of consumer preference for variety. For the Bertrand price to be higher, Bertrand variety (product differentiation) must be significantly greater than Cournot variety. Bertrand consumer surplus falls due to the large increase in the Bertrand price as β increases; but the consumer benefit from greater variety under Bertrand competition is sufficient to ensure that Bertrand consumer surplus never falls below Cournot consumer surplus.

**Fig. 6:**
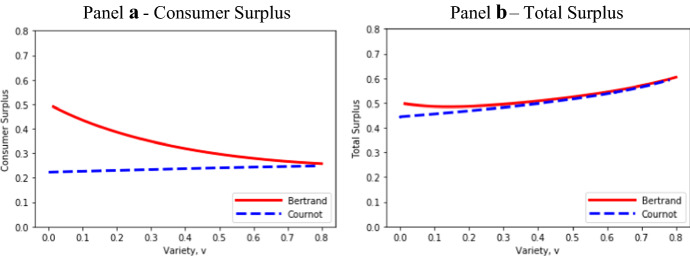
Effects of variety on consumer and total surplus

Figure 6 provides a comparison with exogenous product differentiation by plotting Bertrand and Cournot surplus measures at a common level of variety—*v*—that assumes Bertrand entry: β > 2.09. The basic insight that consumer and total surplus are higher under Bertrand than Cournot competition applies; but if variety is endogenous, the pattern of the relationships shown in Fig. 6 is misleading—most notably due to the omission of the region of Cournot me-too entry.

## Concluding Remarks

One overall theme of our analysis is that the endogeneity of horizontal product differentiation is an important and under-studied consideration in oligopoly theory. We explicitly model an entrant’s decision of whether and how much to differentiate its product horizontally from an incumbent’s. This approach has interesting implications for the comparison of Bertrand and Cournot modes of competition—including implications for consumer welfare.

Not surprisingly, the mode of competition is important for whether entry occurs and for the likelihood and extent of product differentiation by the entrant. A Bertrand firm will not enter with a homogeneous product and therefore stays out if the cost of product differentiation is too high: if investment effectiveness—β—is too low. A Cournot firm always enters and undertakes me-too entry if the cost of differentiation is high and differentiates if it is sufficiently low.

What is surprising is that allowing for endogenous product differentiation can change the conventional wisdom with respect to the comparison of Bertrand and Cournot modes of competition: If the cost of differentiation is positive but sufficiently low—if β is sufficiently high—Bertrand industry profit is equal to or higher than Cournot industry profit, and the Bertrand price may also be higher. These results are due to the much greater marginal value of product differentiation to the Bertrand entrant.

The effects of endogenous product differentiation on consumer surplus are also of interest. An increase in β can arise from an improvement in product differentiation technology that potentially generates surplus to consumers as well as firms. In the Cournot regime, if β is high enough to induce product differentiation, further increases in β increase both industry profit and consumer surplus. However, in the Bertrand case, consumer surplus declines as β increases beyond the critical value for Bertrand entry: Market power is raised by enough to more than offset the benefits to consumers of increased variety. Thus, Bertrand firms get more than 100% of the surplus that is created by improvements in differentiation technology.

Our specific results are of course limited by our model. Other approaches to horizontal differentiation—particularly the Hotelling approach (as in Kishihara & Matsubayashi, 2020)—differ in important respects. And we have abstracted from commonly observed phenomena such as price wars and collusion.

Nevertheless, we believe that incorporating costly endogenous product differentiation into the analysis of entry—and of oligopolistic competition more generally—addresses an empirically significant phenomenon. Moreover, comparisons of Bertrand and Cournot competition arise in many policy applications, such as merger policy, international trade policy, and intellectual property policy. Our analysis suggests that recognizing the endogeneity of product differentiation may significantly affect policy conclusions in these important areas.

## Appendices

### Appendix 1: Bertrand competition

#### **Lemma 1**

*A Bertrand entrant’s stage 1 profit function is strictly concave in k for all* *k* ∈ [0, ∞).

#### *Proof*

From dπ^*j*^(*k*)/d*k* = (d*Φ*^*j*^(*s*)/d*s*)*s*′(*k*) − 1 for (see (8) and (11)), we obtain d^2^π^*j*^(*k*)/(d*k*)^2^ = *s"*(*k*){(d*Φ*^*j*^(*s*)/d*s*) + [(*s'*(*k*))^*2*^*/s"*(*k*)](d^2^*Φ*^*j*^(*s*)/(d*s*)^2^)} for *j* = *B*, *C*. It then follows using *s"*(*k*) = β^2^*s* and (*s'*(*k*))^*2*^*/s"*(*k*) = *s* (see (3) and (4)), that, for both Bertrand and Cournot competition,

A1 $${\text{d}}^{2} \pi ^{j} \left( k \right)/\left( {{\text{d}}k} \right)^{2} = {\upbeta }^{2} s\left[ {{\text{d}}\Phi ^{j} \left( s \right)/{\text{d}}s + s\left( {{\text{d}}^{2} \Phi ^{j} \left( s \right)/\left( {{\text{d}}s} \right)^{2} } \right)} \right].$$

From *Φ*^B^(*s*) = (1 − *s*^2^)(*x*^B^(*s*))^2^ from (5) and d*x*^*B*^(*s*)/ds from (6), it can be shown that

A2 $${\text{d}}\Phi ^{B} \left( s \right)/{\text{d}}s = {-}2\left( {x^{B} \left( s \right)} \right)^{2} \left( {1{-}s + s^{2} } \right)/\left( {2{-}s} \right) < 0.$$

From (A2) using (6) we further obtain

A3 $${\text{d}}^{2} \Phi ^{B} \left( s \right)/\left( {{\text{d}}s} \right)^{2} = 6\left( {x^{B} \left( {\text{s}} \right)} \right)^{2} \left[ {1{-}3s + s^{2} \left( {1{-}s} \right)} \right]/\left[ {\left( {2{-}s} \right)^{2} \left( {1 + s} \right)} \right].$$

If d^2^*Φ*^*B*^(*s*)/(d*s*)^2^ < 0, then, from (A1) and (A2), we obtain d^2^π^*B*^(*k*)/(d*k*)^2^ < 0. But (A3) implies d^2^*Φ*^*B*^(*s*)/(d*s*)^2^ > 0 for *s* ≤ 1/3. Expanding (A1) using (A2) and (A3), we can show that

A4 $${\text{d}}^{2} {\uppi }^{B} \left( k \right)/\left( {{\text{d}}k} \right)^{2} = {-}2{\upbeta }^{2} s\left( {x^{B} \left( {\text{s}} \right)} \right)^{2} {\text{Z}}^{B} /\left[ {\left( {2{-}s} \right)^{2} \left( {1 + ~s} \right)} \right],$$

where Z^*B*^ ≡ (1 − *s* + *s*^2^)(2 − *s*)(1 + *s*) − 3*s*(1 − 3*s* + *s*^2^(1 − *s*)). With further manipulation, we obtain Z^*B*^ = 2(1 − *s*)(1 − *s* + *s*^2^) + *s*^2^(5 + *s* + 2*s*^2^) and hence Z^*B*^ > 0 for *s* ∈ [0, 1] and, from (A4), d^2^π^*B*^(*k*)/(d*k*)^2^ < 0 for *s* ∈ (0, 1]. From *s* = e^−β*k*^, profit is strictly concave for *k* ∈ [0, ∞).

#### *Proof of Proposition 2*

(Characteristics of Bertrand investment and entry)

(i) Due to *E* > 0, Bertrand entry requires investment as proved in the text. To show that investment, *k* = *k*^*B*^ is unique and finite if entry takes place, we use (8) and *s*'(*k*) =  − β*s*(*k*) (see (4)) to obtain dπ^*B*^(*k*)/d*k* =  − 2β*s*(*k*)(d*Φ*^*B*^(*s*)/d*s*) − 1. It then follows from lim_k→∞_ *s*(*k*) = 0 (see (3)) and d*Φ*^*B*^(*s*)*/*d*s* as in (A2) that lim_k→∞_ dπ^*B*^(*k*)/d*k* =  − 1: infinite investment reduces profit, so *k*^*B*^ is finite. From (8) and *k*^*B*^ > 0, we obtain dπ^*B*^(0)/d*k* > 0 and, using d^2^π^*B*^(*k*)/(d*k*)^2^ < 0 for *k* ∈ [0, ∞) (see Lemma 1), it follows that *k*^*B*^ satisfies dπ^*B*^(*k*)/d*k* = 0 and is unique.

(ii) From part (i), investment is necessary for Bertrand entry. To show that *β* > *2/*(*a* − *c*)^*2*^ is necessary for entry, we show that it is necessary for investment. From (8) using d*Φ*^*B*^(1)/d*s* =  − 2(*x*^*B*^(1))^2^ =  − (*a* − *c*)^2^/2 (see (A2) and (5)) and *s*′(0) =  − β (see (4)), we obtain *k*^*B*^ > 0 if and only if dπ^*B*^(0)/d*k* = (d*Φ*^*B*^(1)*/*d*s*)*s*′(0) − 1 = β(*a* − *c*)^2^/2 − 1 > 0, which proves the result.

(iii) For β = 2.09/(*a* − *c*)^2^, we use dπ^*B*^(*k*)/d*k* = 0 (see (8)), (A2) and (5) to solve for *s* = *s*(*k*^*B*^) = 0.989, which, from *k* =  − (ln *s*)/β (see (3)), implies *k*^*B*^ = 0.005. Using these values and *E* = 0.0001(*a* − *c*)^2^ (from (9)) in (7), we obtain π^*B*^(*k*^*B*^) = 0. Since dπ^j^(*k*^*j*^)/dβ > 0 (shown subsequently in Proposition 5), Bertrand entry occurs if and only if β ≥ β^E^ ≡ 2.09/(*a* − *c*)^2^.

### Appendix 2: Cournot Competition

#### **Lemma 2**

*A Cournot entrant’s stage-1 profit function is strictly concave in k for all k* ∈ [0, ∞).

#### *Proof*

From *Φ*^*C*^(s) = (*x*^*C*^(*s*))^*2*^ and x^C^(*s*) = (*a* − *c*)/(2 + *s*) (see (10)), we obtain

A5 $${\text{d}}\Phi ^{C} \left( s \right)/{\text{d}}s = {-}2\left( {x^{C} \left( s \right)} \right)^{2} /\left( {2 + s} \right) < 0.$$

Differentiating (A5) yields d^2^*Φ*^*C*^(*s*)/(d*s*)^2^ = 6(*x*^*C*^(*s*))^2^/(2 + *s*)^2^ and hence from (A1)

A6 $${\text{d}}^{2} {\uppi }^{C} \left( k \right)/\left( {{\text{d}}k} \right)^{2} = - 4{\upbeta }^{2} s\left( {1{-}s} \right)\left( {x^{C} \left( s \right)} \right)^{2} /\left( {2 + s} \right)^{2} < 0,$$

for all *s* ∈ (0, 1] and all *k* ∈ [0, ∞).

#### *Proof of Proposition 3*

(Characteristics of Cournot investment and entry)

(i) From the text, entry is profitable even without investment. To show that *k* = *k*^C^ is finite and unique, from (11) using (4) and (A5), we obtain dπ^*C*^(*k*)/d*k* = [2β*s*(*k*)(*x*^*C*^(*s*))^2^/(2 + *s*)] − 1. It follows using lim_k→∞_ *s*(*k*) = 0 (see (3)) that lim_k→∞_ dπ^*C*^(*k*)/d*k* =  − 1: infinite investment reduces profit so *k*^*C*^ is finite. If dπ^*C*^(0)/d*k* ≤ 0, then *k*^*C*^ = 0 from (11). If dπ^*C*^(0)/d*k* > 0, then *k*^*C*^ > 0 and it follows using d^2^π^*C*^(*k*)/(d*k*)^2^ < 0 for *k* ∈ [0, ∞) (see Lemma 2) that *k*^*C*^ satisfies dπ^*C*^(*k*)/d*k* = 0 and is unique.

(ii) To show *k*^*C*^ = 0 for β ≤ 13.5/(*a* − *c*)^2^ and *k*^*C*^ > 0 for β > 13.5/(*a* − *c*)^2^, we use d*Φ*^*C*^(1)/d*s* =  − 2(*x*^*C*^(1))^2^/3 =  − 2(*a* − *c*)^2^/27 (from (A5) and (10)) and *s*′(0) =  − β in (11) to obtain dπ^*C*^(0)/d*k* = 2β(*a* − *c*)^2^/27 − 1. From (11), we have *k*^*C*^ > 0 if and only if dπ^*C*^(0)/d*k* > 0 and the results follow.

### Appendix 3: Investment Comparisons and the Effects of β

#### *Proof of Proposition 4*

(Bertrand and Cournot investment comparisons)

(i) β ≤ 2/(*a* − *c*)^2^, then *k*^*B*^ = *k*^*C*^ = 0 from Propositions 2(ii) and 3(ii).

(ii) If β^E^ ≤ β ≤ 13.5/(*a* − *c*)^2^, then *k*^*B*^ > *k*^*C*^ = 0 from Propositions 2(iii) and 3(ii).

For β = 13.5/(*a* − *c*)^2^, we use dπ^B^(*k*)/d*k* = 0 (see (8)), (A2) and (5) to solve for *s*(*k*^*B*^) = 0.3918 and *v*^*B*^ = 0.6082. Since *v* = 0 at β = 0 and *v* → 1 in the limit as β → ∞, it follows that *v*^*B*^ = 0.608 represents 60.8% of the potential product differentiation as measured by *v*.

(iii) If β > 13.5/(*a* − *c*)^2^ then *k*^*B*^ > 0 and *k*^*C*^ > 0 from Propositions 2(iii) and 3(ii). It follows from strict concavity of π^*B*^(*k*) (Lemma 1) and from dπ^*B*^(*k*^*B*^)/d*k* = 0, that *k*^*B*^ > *k*^*C*^ if and only if dπ^*B*^(*k*^*C*^)/d*k* > 0. From (8), *s*′(*k*) =  − β*s* and d*Φ*^*B*^(*s*)/d*s* as in (A2)), we obtain dπ^*B*^(*k*^*C*^)/d*k* = [2β*s*^*C*^(*x*^B^(*s*^*C*^))^2^(1 − *s*^*C*^ + (*s*^*C*^)^2^)/(2 − *s*^*C*^)] − 1. To show dπ^*B*^(*k*^*C*^)/d*k* > 0, from (11), (A5) and *s*'(*k*) =  − β*s* (see (4)), we obtain dπ^*C*^(*k*^*C*^)/d*k* = [2βs^C^(*x*^*C*^(s^C^))^2^/(2 + *s*^C^)] − 1 = 0. Using *x*^*B*^(*s*) = (2 + *s*)*x*^*C*^(*s*)/(2 + *s* − *s*^2^) > *x*^*C*^(*s*) (from (5) and (10)), we then obtain 2β*s*^*C*^(*x*^*B*^(*s*^C^))^2^ > 2 + *s*^*C*^. Hence dπ^*B*^(*k*^*C*^)/d*k* > [(2 + *s*^*C*^)(1 − *s*^C^ + (*s*^*C*^)^2^)/(2 − *s*^*C*^)] − 1 = (*s*^*C*^)^2^(1 + *s*^*C*^)/(2 − *s*^*C*^) > 0, which implies *k*^*B*^ > *k*^*C*^.

#### *Proof of Proposition 5*

(Effects of an increase in *β*)

(i), For *k*^*j*^ > 0, to show that *k*^*j*^ initially increases and then declines in β, we use dπ^*j*^(*k*^*j*^)/d*k* = 0 for *j* = *B*, *C*, to obtain d*k*^*j*^/dβ =  − (∂^2^π^j^/(∂*k*^*j*^)(∂β))/(d^2^π^*j*^(*k*^*j*^)/(d*k*^*j*^)^2^). From dπ^*j*^(*k*)/d*k* = (d*Φ*^*j*^(*s*)/d*s*)s′(*k*) − 1 (see (8) and (11)), *s*′(*k*) =  − β*s* and ∂*s*/∂β =  − *ks* (see (3)), we obtain

A7 $$\partial ^{2} \pi ^{j} /\left( {\partial k} \right)\left( {\partial \beta } \right) = ~{-}s\left( {{\text{d}}\Phi ^{j} /{\text{d}}s} \right) + k^{j} {\upbeta }s\left[{\text{d}}\Phi ^{j} /{\text{d}}s + s{{\text{d}}^{2} \Phi ^{j} /\left( {{\text{d}}s} \right)^{2} } \right].$$

Since d*k*^*j*^/dβ =  − ∂^2^π^*j*^/(∂*k*)(∂β)/(d^2^π^*j*^(*k*)/(d*k*)^2^) it follows from (A7) and (A1) that

A8 $${\text{d}}k^{j} /{\text{d}}{\upbeta } = \left\{ {{-}k^{j} + \left[ {\left( {{\text{d}}\Phi ^{j} /{\text{d}}s} \right)/{\upbeta }\left( {{\text{d}}\Phi ^{j} /{\text{d}}s + s{\text{d}}^{2} \Phi ^{j} /\left( {{\text{d}}s} \right)^{2} } \right)} \right]} \right\}/{\upbeta }.$$

The second term of (A8) is positive for *j* = *B*, *C* due to d*Φ*^*j*^(*s*)/d*s* < 0 (see (A2) and (A5)) and d^2^π^*j*^(*k*)/(d*k*)^2^ < 0 (see Lemmas 1 and 2). If β is small, then the second term of (A8) dominates, and d*k*^*j*^/dβ > 0, but for β sufficiently large, the negative first term dominates, and d*k*^*j*^/dβ < 0.

(ii) To show that product differentiation is increasing in β, from d*v*(*k*^*j*^;β)/dβ = ∂*v*/∂β + *v*′(*k*^*j*^)(d*k*^*j*^/dβ) where ∂*v*/∂β = *ks* and *v*′(*k*) = β*s* from (4), we obtain d*v*(*k*^*j*^)/dβ = *s*[*k*^*j*^ + β(d*k*^*j*^/dβ)]. We then substitute for d*k*^*j*^/dβ from (A8)), use d*Φ*^*j*^/d*s* < 0 (from (A2) and (A5)), and use (A1) and Lemmas 1 and 2 to show that for *k*^*j*^ > 0 and *j* = *B*, *C*,

A9 $${\text{d}}v\left( {k^{j} ;{\upbeta }} \right)/{\text{d}}{\upbeta } = s({\text{d}}\Phi ^{j} \left( s \right)/{\text{d}}s)/{\upbeta }\left[ {{\text{d}}\Phi ^{j} \left( s \right)/{\text{d}}s + s{\text{d}}^{2} \Phi ^{j} /\left( {{\text{d}}s} \right)^{2} } \right] > 0.$$

(iii) With respect to prices, from (5), (10), and *v* = 1 − *s*, we obtain

A10 $${\text{d}}p^{\beta } \left( s \right)/{\text{d}}v = \left( {a{-}c} \right)/\left( {2{-}s} \right)^{2} ;~\quad {\text{d}}p^{C} \left( s \right)/{\text{d}}v = \left( {a{-}c} \right)/\left( {2 + s} \right)^{2} .$$

Hence from (A10) and (A9), d*p*^*j*^(*s*)/dβ = (d*p*^*j*^(*s*)/d*v*)(d*v*(*k*^*j*^)/dβ) > 0 for *k*^*j*^ > 0.

With respect to profits, the entrant’s profit is increasing in β: from dπ^*j*^(*k*^*j*^;β)/dβ = (dπ^*j*^(*k*)/d*k*)(d*k*^*j*^/dβ) + ∂π^j^/∂β, it follows using dπ^*j*^(*k*)/d*k* = 0 for *k*^*j*^ > 0 (see (8) and (11)), ∂*s*^*j*^/∂β =  − *k*^*j*^*s*^*j*^ < 0 and d*Φ*^*j*^(*s*)/d*s* < 0 (see (A2) and (A5)) that for *j* = *B*, *C*,

A11 $${\text{d}}{\uppi }^{{\text{j}}} \left( {k^{j} ;{\upbeta }} \right)/{\text{d}}{\upbeta } = \partial {\uppi }^{{\text{j}}} \left( {k^{j} } \right)/\partial {\upbeta } = - k^{j} s^{j} \left( {{\text{d}}\Phi ^{j} \left( s \right)/{\text{d}}s} \right) > 0.$$

The incumbent’s profit (variable profit) is also increasing in β: from d*Φ*^*j*^(*s*)/dβ = (d*Φ*^*j*^(*s*)/d*s*)(d*s*^*j*^/dβ), it follows using d*s*^*j*^/dβ = d*s*(*k*^*j*^;β)/dβ =  − d*v*(*k*^*j*^;β)/dβ < 0 from (A9) and d*Φ*^*j*^(*s*)/d*s* < 0 (see (A2) and (A5)), that d*Φ*^*j*^(*s*)/dβ > 0 for *j* = *B*, *C*.

### Appendix 4: Lemma 3

#### **Lemma 3**

*Normalization with respect to parameter values, a and c.*

*Let superscript 0 denote variables evaluated at a − c = 1. For Bertrand and Cournot competition:*

(i) *If k* ^*j*^ *> 0, the degree of substitutability, s* ^*j*^ *for j = B, C, depends only on γ ≡ β(a − c)* ^*2*^ *.*
(ii) *If β = γ* ^*0*^ */(a − c)* ^*2*^ *where a − c > 0, the degree of substitutability and the thresholds for investment are the same as if a − c = 1. Investment and profit are increased by the factor (a − c)* ^*2*^ *.*

#### *Proof*

(i) If *k*^*C*^ > 0, it follows from (11), (A5), (10) and (4), that dπ^*C*^(*k*^*C*^)/d*k* = [γ*s*^*C*^/(2 − *s*^*C*^)^3^] − 1 = 0, which proves that *s*^*C*^ ≡ *s*(*k*^*C*^) depends only on γ ≡ β(*a* − *c*)^2^. Similarly, if *k*^*B*^ > 0, it follows from dπ^*B*^(*k*^*B*^)/d*k* = 0 using (8), (A2), (5) and (4) that *s*^*B*^ ≡ *s*(*k*^*B*^; β) depends only on γ. We denote the functions relating *s*^j^ to γ as *s*^j^ = g^j^(γ) for j = B, C.

(ii) Propositions 1 and 2 imply *k*^*B*^ > 0 iff β ≥ β^*E*^ **≡** 2.09/(*a* − *c*)^2^ and *k*^*C*^ > 0 iff β > 13.5/(*a* − *c*)^2^. If β = γ^0^/(*a* − *c*)^2^, then *k*^*B*^ > 0 iff γ^0^ ≥ 2.09 and *k*^*C*^ > 0 iff γ^0^ > 13.5, which are the respective thresholds if *a* − *c* = 1. Also, if β = γ^0^/(*a* − *c*)^2^ then *s*^*j*^ = g^*j*^(γ^0^) (see part (i)) proof), which is the value of *s*^*j*^ at *a* − *c* = 1. From *s* = e^−β*k*^ (see (3)) and β = γ^0^/(*a* − *c*)^2^, we obtain *k*^j^ =  − ln{g^j^(γ^0^)}/β. If *a* − *c* = 1, then β^0^ = γ^0^ and *k*^j0^ =  − ln{g^j^(γ^0^)}/γ^0^. Hence

A12 $$~k^{{\text{j}}} = - {\text{ln}}\left\{ {{\text{g}}^{{\text{j}}} \left( {\gamma ^{0} } \right)} \right\}/{\upbeta } = \left( {a{-}c} \right)^{2} k^{{{\text{j}}0}} \quad {\text{for}}\quad j = B,C.$$

From (10), we obtain *Φ*^C^ = (*a* − *c*)^2^/(2 + g^*C*^(γ^0^)^2^) = (a − c)^2^*Φ*^*C*0^ and from (5), *Φ*^B^ = (*a* − *c*)^2^*Φ*^B0^. Hence for β = γ^0^/(*a* − *c*)^2^ it follows using (A12) that, for *j* = *B*, *C*,

A13 $${\uppi }^{{\text{j}}} \left( {k^{{\text{j}}} } \right) = \left( {a{-}c} \right)^{2} \left[ {\Phi ^{{{\text{j}}0}} {-}k^{{{\text{jo}}}} {-}0.0001} \right] = \left( {a{-}c} \right)^{2} {\uppi }^{{{\text{j}}0}} \left( {k^{{{\text{j}}0}} } \right).$$

### Appendix 5: Output and Price Comparisons

#### *Proof of Proposition 6*

(Bertrand and Cournot output comparison).

From (5) and (10), the difference between Bertrand and Cournot output is

A14 $$x^{B} \left( {s^{B} } \right) - x^{C} \left( {s^{C} } \right) = \left( {a{-}c} \right)\left[ {s^{C} {-}s^{B} + \left( {s^{B} } \right)^{2} } \right]/\left( {2{-}s^{B} } \right)\left( {1 + s^{B} } \right)\left( {2 + s^{C} } \right).$$

If there is Bertrand entry (β ≥ β^E^), then *k*^*B*^ > *k*^*C*^ (see Proposition 4), which, using *s*′(*k*) =  − β*s* < 0, implies *s*^*C*^ > *s*^*B*^ and hence *x*^*B*^(*s*^*B*^) > *x*^*C*^(*s*^*C*^).

#### *Proof of Proposition 7*

(Bertrand and Cournot price comparison)

To show that *p*^*B*^ > *p*^*C*^ for *β* ∈ (10.125/(*a* − *c*)^2^, 15.25/(*a* − *c*)^2^], we first recall from the text (see (13) that if *k*^*C*^ = 0 and s^C^ = 1 (Cournot me-too entry), then *p*^*B*^ > *p*^*C*^ if and only if *v*^*B*^ ≡ 1 − *s*^*B*^ > ½. From (5), (10), and dπ^*B*^(*k*)/d*k* = 0 (see (8)), we find that if β = 10.125/(*a* − *c*)^2^, then

A15 $$s^{B} = v^{B} = 1/2\quad {\text{and}}\quad p^{B} = p^{C} = \left( {a{-}c} \right)/3 + c.$$

If β = 13.5/(*a* − *c*)^2^, then *v*^B^ = 0.608 from Proposition 4 and, using *s*^*B*^ = 0.392 in (5), we obtain *p*^*B*^ = 0.378(*a* − *c*) + *c*. Since d*p*^*B*^/dβ > 0 for *k*^*B*^ > 0 (Proposition 5(iii)) and *p*^*C*^ is constant when *k*^*C*^ = 0, it follows using (A15) and Proposition 3 that *p*^*B*^ > *p*^*C*^ and *v*^*B*^ ∈ (0.5, 0.608] for β ∈ (10.125/(*a* − *c*)^2^, 13.5/(*a* − *c*)^2^].

If β > 13.5/(*a* − *c*)^2^, then *k*^*C*^ > 0 and d*p*^*C*^/dβ > 0 (Proposition 5(iii)), but *p*^*B*^ > *p*^*C*^ for some initial range of β > 13.5/(*a* − *c*) due to the continuity of *p*^*B*^ and *p*^*C*^ in β. Specifically, if β = 15.25/(*a* − *c*)^2^, then *s*^*C*^ = 0.5326 and *s*^*B*^ = 0.3474 (from (11) and (8)) so *s*^*B*^ < *s*^*C*^/(1 + *s*^*C*^) = 0.3475 and *p*^*B*^ > *p*^*C*^ from (13). Thus we have *p*^*B*^ > *p*^*C*^ for β ∈ (13.5/(*a* − *c*)^2^, 15.25/(*a* − *c*)^2^] as claimed. If β = 15.26/(*a* − *c*)^2^, then *s*^*C*^ = 0.5317 and *s*^*B*^ = 0.3472 > *s*^*C*^/(1 + *s*^*C*^) = 0.3471, so *p*^*B*^ < *p*^*C*^. If β = 15.257/(*a* − *c*)^2^, then *s*^*C*^ = 0.5320 and *s*^*B*^ = 0.3473 = *s*^*C*^/(1 + *s*^*C*^), so *p*^*B*^ = *p*^*C*^.

### Appendix 6: Profit and Surplus Comparisons

#### *Proof of Proposition 8*

(Bertrand and Cournot profit comparison)

(i) To show that variable profit is higher under Bertrand than Cournot competition if *k*^*C*^ > 0 or β > 13.5/(*a* − *c*)^2^ (see Proposition 2), we first use (5) and (10) to obtain the difference in variable profit: *Φ*^*B*^(*s*^*B*^) − *Φ*^*C*^(*s*^*C*^) = (*a* − *c*)^2^Ω/(2 − *s*^*B*^)^2^(1 + *s*^*B*^)(2 + *s*^*C*^)^2^ where

A16 $$\Omega \equiv \left( {1{-}s^{B} } \right)\left( {2 + s^{C} } \right)^{2} {-}\left( {1 + s^{B} } \right)\left( {2{-}s^{B} } \right)^{2} .$$

From *k*^*C*^ > 0 and β > 13.5/(*a* − *c*)^2^, we have *k*^*B*^ > 0 (see Proposition 3); and it follows from the first-order conditions, dπ^*C*^(*k*)/d*k* = dπ^*B*^(*k*)/d*k* = 0 (see (8) and (11)), and from (A2), (A5), (5), (10) and (4) that *s*^*C*^(*x*^*C*^(*s*^*C*^))^2^/(2 + *s*^*C*^) = *s*^*B*^(*x*^*B*^(*s*^*B*^))^2^(1 − *s*^*B*^ + (*s*^*B*^)^2^)/(2 − *s*^*B*^) = 1/(2β) and hence that

A17 $$s^{C} /\left( {2 + s^{C} } \right)^{3} ~ = ~s^{B} \left( {1{-}s^{B} + \left( {s^{B} } \right)^{2} } \right)/\left( {1 + s^{B} } \right)^{2} \left( {2{-}s^{B} } \right)^{3} .$$

Using (A17) to substitute for the 2^nd^ term of (A16), we obtain Ω = (2 + *s*^*C*^)^2^[*s*^*C*^(1 − (*s*^*B*^)^2^)(2 − *s*^*B*^) − *s*^*B*^(1 − *s*^*B*^ + (*s*^*B*^)^2^)(2 + *s*^*C*^)]/*s*^*C*^(1 + *s*^*B*^)(2 − *s*^*B*^). If we gather terms in *s*^*C*^, it can be shown that

A18 $$\Omega = 2\left( {2 + s^{C} } \right)^{2} \left[ {s^{C} \left( {1{-}s^{B} - \left( {s^{B} } \right)^{2} /2} \right) {-}s^{B} \left( {1{-}s^{B} + \left( {s^{B} } \right)^{2} } \right)} \right]/s^{C} \left( {1 + s^{B} } \right)\left( {2{-}s^{B} } \right).$$

To show that *Φ*^*B*^(*s*^*B*^) − *Φ*^*C*^(*s*^*C*^) > 0 for *k*^*C*^ > 0, it remains to show that Ω > 0 for all β > 13.5/(*a* − *c*)^2^. Using *s*^*B*^ = 1 − *v*^*B*^ = 0.392 (see Proposition 4(ii)), *s*^*C*^ = 1, (5) and (10), we obtain *Φ*^*B*^(*s*^*B*^) − *Φ*^*C*^(*s*^*C*^) = 0.203 > 0 at β = 13.5/(*a* − *c*)^2^. Hence, from the continuity of Ω in β, the possibility that Ω < 0 arises only if Ω = 0 at some finite value of β > 13.5/(*a* − *c*)^2^.

We next show that Ω = 0 implies a contradiction. If Ω = 0, then, from (A18),

A19 $$s^{C} = s^{B} \left( {1{-}s^{B} + \left( {s^{B} } \right)^{2} } \right)/{\text{D}}\,\,\,\,{\text{where}}\,\,\,{\text{D}} \equiv 1{-}s^{B} - \left( {s^{B} } \right)^{2} /2.$$

From (A19), we obtain 2 + *s*^*C*^ = (1 − (*s*^*B*^)^2^)(2 − *s*^*B*^)/D and hence that *s*^*C*^/(2 + *s*^*C*^)^3^ = *s*^*B*^(1 − *s*^*B*^ + (*s*^*B*^)^2^)D^2^/(1 − (*s*^*B*^)^2^)^3^(2 − *s*^*B*^)^3^. Using (A17) to cancel terms, we further obtain D^2^ = (1 − (*s*^*B*^)^2^)^3^/(1 + *s*^*B*^)^2^ = (1 − (*s*^*B*^)^2^)(1 − s^B^)^2^. When we expand D from (A19), the expression reduces to (*s*^*B*^)^3^((5/4)*s*^*B*^ − 1) = 0, which implies *s*^*B*^ = 4/5 or *s*^*B*^ = 0. If *s*^*B*^ = 4/5, then D =  − 0.12 and *s*^*C*^ =  − 5.6, which violates s^C^ > 0. Consequently, the possibility that *s*^*B*^ = *s*^*C*^ = 0 applies only in the limit as β → ∞. Thus Ω > 0 for all finite values of β > 13.5/(*a* − *c*)^2^, which proves the result.

(ii) To show Bertrand industry profit equals or exceeds Cournot industry profit, letting π^jT^(k^j^) for *j* = B, C (T for total) denote equilibrium industry profit under Bertrand and Cournot competition respectively, then using (A13), we obtain π^jT^(*k*^j^) = (*a* − *c*)^2^[2*Φ*^j0^ − *k*^jo^ − 0.0001] = (*a* − *c*)^2^π^jT0^(*k*^j0^), where the superscript 0 denotes values when *a* − *c* = 1. Consequently, π^BT^(*k*^*B*^) − π^CT^(*k*^*C*^) = (*a* − *c*)^2^[π^BT0^(*k*^B0^) − π^CT0^(*k*^C0^)]. Our simulation with *a* − *c* = 1 and β > 13.5, shows that π^BT0^(*k*^B0^) − π^CT0^(*k*^C0^) ≥ 0 and hence π^BT^(*k*^*B*^) − π^CT^(*k*^*C*^) ≥ 0.

#### *Proof of Proposition 9*

(Effects of β on consumer and total surplus)

(i) For Bertrand competition, entry takes place and *k*^*B*^ > 0 for β ≥ β^*E*^ **≡** 2.09/(*a* − *c*)^2^ from Proposition 2. To show that consumer surplus is decreasing in β for *k*^*B*^ > 0, we first use *G*^*B*^(*s*) = (1 + *s*)(*x*^*B*^(*s*))^2^ from (14), and d*x*^*B*^(*s*)/d*s* from (6), to obtain

A20 $${\text{d}}G^{B} \left( s \right)/{\text{d}}s = 3s\left( {x^{B} \left( s \right)} \right)^{2} /\left( {2{-}s} \right) > 0.$$

From d*s*(*k*^*B*^;β)/dβ =  − d*v*(*k*^*B*^;β)/dβ < 0 (see (A9)), we obtain d*G*^*B*^(*s*)/dβ = (d*G*^*B*^(*s*)/d*s*)(d*s*^*B*^/dβ) < 0.

We denote total surplus by *S*^*Tj*^ ≡ *G*^*j*^(*s*^*j*^) + *Φ*^j^(*s*^*j*^) + π^j^(*k*^*j*^;β). To show that d*S*^*TB*^/dβ > 0 for *s*^*B*^ ≤ 1/2 or *v*^*B*^ ≥ ½, which, from (A15), applies if β ≥ 10.125/(*a* − *c*)^2^, we use (A20) and (A2), to obtain d*S*^*TB*^/dβ =  − (*x*^*B*^(*s*^*B*^))^2^(1 − 2*s*^*B*^)(d*s*^*B*^/dβ) + dπ^*B*^(*k*^*B*^;β)/dβ. The result follows using d*s*^*B*^/dβ < 0 from (A9) and dπ^*B*^(*k*^*B*^;β)/dβ > 0 from (A11).

(ii) For Cournot competition, entry does not require investment, but β has no effect unless *k*^*C*^ > 0, which applies if β > 13.5/(*a* − *c*)^2^ (see Proposition 3). To show that consumer surplus is increasing in β for *k*^*C*^ > 0, we use (14) to obtain d*G*^*C*^(*s*)/d*s* =  − *s*(*x*^*C*^(*s*))^2^/(2 + *s*) < 0. Using d*s*^*C*^/dβ =  − d*v*^*C*^(k^*C*^;β)/dβ < 0 from (A9), it follows that d*G*^*C*^(*s*)/dβ = (d*G*^*C*^(*s*)/d*s*)(d*s*^*C*^/dβ) > 0 for β > 13.5/(*a* − *c*)^2^. Both the incumbent’s profit and the entrant’s profit are increasing in β (see Proposition 5), so total surplus is also increasing in β.

(iii) To show that for a given value of β, consumer surplus is higher under Bertrand than Cournot competition, using *G*^*j*^(*s*) = (1 + *s*)(*x*^*j*^(*s*))^2^ for *j* = B,C (see (14)), *x*^*B*^(*s*) = (*a* − *c*)/(2 − *s*)(1 + *s*) (see (5)) and *x*^*C*^(*s*) = (*a* − *c*)/(2 + *s*) (see (10)), we first show that

A21 $$G^{B} \left( {s^{B} } \right) {-}G^{C} \left( {s^{C} } \right) = \left( {a{-}c} \right)^{2} {\text{H}}/\left( {2{-}s^{B} } \right)^{2} \left( {1 + s^{B} } \right)\left( {2 + s^{C} } \right)^{2} ,$$

where H ≡ (2 + *s*^*C*^)^2^ − (1 + *s*^*C*^)(2 − *s*^*B*^)^2^(1 + *s*^*B*^). Expanding H using (2 − *s*^*B*^)(1 + *s*^*B*^) = 2 + *s*^*B*^ − (*s*^*B*^)^2^, we obtain H = (2 + *s*^*C*^)^2^ − (1 + *s*^*C*^)(2 − *s*^*B*^)(2 + *s*^*B*^) + (1 + *s*^*C*^)(2 − *s*^*B*^)(*s*^*B*^)^2^ which reduces to H ≡ (*s*^*C*^)^2^ + (1 + *s*^*C*^)(3 − *s*^*B*^)(*s*^*B*^)^2^ > 0 and hence *G*^*B*^(*s*^*B*^) − *G*^*C*^(*s*^*C*^) > 0 for any *s*^*j*^ ∈ (0,1].

If k^C^ > 0, then Bertrand industry profit equals or exceeds Cournot industry profit (see Proposition 8(ii)) and total surplus is higher under Bertrand than Cournot competition.

## Footnotes

1 Common examples of “me too” products include house brands and generic drugs that seem to be based on lower marginal cost, weaker branding, and/or on free-riding on the national brands’ category development investments.

2 Slade (1995) summarizes empirical work that uses Cournot and Bertrand models and reports no cases that apply the Bertrand model to a homogeneous product. The Cournot model is commonly applied in such cases.

3 Our price comparison is not an “apples to apples” comparison as a Bertrand entrant will enter with a more differentiated product than would a Cournot entrant. Even so, the insight that product differentiation under Bertrand competition can be sufficient to raise the Bertrand price above the Cournot price is interesting in itself and also helps explain our profit results.

4 Brander and Spencer (2015a) examine simultaneous investment in product differentiation. Most insights from the current version extend to that setting. The conditions that determine the decision to invest are the same. Our results are more transparent and more easily developed in the context of entry.

5 In contrast, the Hotelling location-based version of product differentiation does not normally yield U-shaped output effects as differentiation (distance between firms) increases. If each consumer can purchase just one unit and the market is “covered” (all consumers are served), then output is constant so there are no output effects. Alternatively, if transport costs are high enough that the market is not covered, output effects are possible. The pattern of output effects depends on the distribution of consumers, the pattern of transport costs, and nature of firm interactions and other model specifics. However, the standard result would be for output effects to be the opposite of U-shaped as differentiation increases. For example, suppose that two firms start at the midpoint of a line segment with a uniform distribution of consumers and with unserved consumers near each endpoint. Initially, as the firms differentiate (move apart), more consumers would be served and output would rise. However, as the firms get further apart, the endpoints would eventually be covered, and an uncovered market segment would emerge between the two firms. Further differentiation would reduce market coverage and output would fall.

6 For each β, the Bertrand simulation for *a* = 1 and *c* = 0 solves numerically for the value of *k* = *k*^*B*^ that maximizes π^*B*^(*k*) *≡* (1 *– s*^2^)/(1 + *s*)^2^(2—*s*)^2^ *– k –* 0.0001 (see (7) and (5)) – subject to *s* = e^−β*k*^. The Cournot simulation solves for *k* = *k*^*C*^ to maximize π^*C*^(*k*) = 1/(2 + *s*)^2^ – *k* – 0.0001*.*

7 From (A2), (A5) and (A10), d*Φ*^*B*^(1)/d*v* = (*a* – *c*)^2^/2, d*Φ*^*C*^(1)/d*v* = (a – c)^2^/13.5, d*p*^*B*^(1)/d*v* = *a*—*c* and d*p*^*C*^(1)/d*v* = (a – c)/9.
